# Job Satisfaction of Nurses in the Context of Clinical Supervision: A Systematic Review

**DOI:** 10.3390/ijerph21010006

**Published:** 2023-12-20

**Authors:** Ali Hudays, Faye Gary, Joachim G. Voss, Ahmed Hazazi, Amal Arishi, Fatimah Al-sakran

**Affiliations:** 1 Frances Payne Bolton School of Nursing, Case Western Reserve University, Cleveland, OH 44106, USA; fxg21@case.edu (F.G.); jgv20@case.edu (J.G.V.); 2 Community, Psychiatric, and Mental Health Nursing Department, College of Nursing, King Saud University, Riyadh 11543, Saudi Arabia; falsakran@ksu.edu.sa; 3 Department of Public Health, Faculty of Health Science, Saudi Electronic University, Riyadh 13316, Saudi Arabia; a.hazazi@seu.edu.sa; 4 Medical Surgical Department, College of Nursing, King Saud University, Riyadh 11543, Saudi Arabia; aarishi@ksu.edu.sa

**Keywords:** clinical supervision, job satisfaction, stress, burnout, registered nurses, psychiatric nurses

## Abstract

The purpose of this systematic review is to gather and analyze data from existing research on the effects of clinical supervision (CS) intervention on nurses’ job satisfaction and related outcomes such as stress levels, burnout, and care quality. Using the PRISMA (preferred reporting items for systematic reviews and meta-analysis) criteria, a systematic review of the research available in the databases PubMed, PsycInfo, Cochrane Library, and CINAHL, well as Google Scholar, between January 2010 and May 2023 was carried out. Out of the 760 studies assessed, only 8 met the criteria for inclusion in the review based on Hawker’s assessment tool. The results indicate that CS has a positive impact on nurses’ job satisfaction and related outcomes such as reduced burnout, stress levels, and the quality of care. The study also found that the effectiveness of CS in enhancing job satisfaction was most evident during the 6-month follow-up period. However, nurses who did not receive CS did not show any noticeable improvement in their knowledge or practice. Additionally, nurses who required more efficient clinical oversight reported little to no positive impact on their practice or training. The review also highlighted gaps in knowledge regarding the frequency and number of sessions required for the impact of CS on nurses’ job satisfaction and other outcomes. Due to the limited number of studies included in this review, further research is recommended to evaluate the influence of CS on nurses’ job satisfaction.

## 1. Introduction

Clinical supervision (CS) is a way of enhancing education and training programs that emphasizes building relationships, focusing on work-related aspects and encompassing activities such as managing, supporting, developing, and evaluating the work of colleagues [1]. Supervision is frequently observed in community mental health settings and other clinical locations, such as counseling centers and hospitals [2,3,4], where a significant portion (54–75%) of practitioners receive 30–60 min of weekly supervision [5,6]. Clinical supervision is a crucial component of professional development and support in the field of health care, particularly in clinical settings [7]. Clinical supervision involves a collaborative relationship between a more experienced and knowledgeable supervisor and a less experienced supervisee, with the goal of enhancing the supervisee’s clinical skills, knowledge, and overall professional competence [8]. The literature on CS offers several classical works that have contributed to defining and exploring the dimensions of this phenomenon in nursing. These works include models of CS such as Proctor’s framework, the seven-eyed model of supervision, and Carroll’s model [9,10,11].

Proctor’s framework of CS outlines the ways in which health-care practitioners can receive support in the formative, restorative, and normative aspects of their profession [11]. The formative aspect pertains to the acquisition and improvement of skills that are relevant to the specific role of health-care professionals, and the restorative aspect involves offering support to professionals in dealing with the emotional challenges associated with their work. The normative aspect relates to ensuring that health-care professionals adhere to standards of care and follow organizational policies and procedures [11]. Therefore, it is essential that there is provision of comprehensive support in CS across Proctor’s three domains to effectively assist allied health professionals [12].

The seven-eyed model of supervision was developed by Hawkins and Shohet in 1989. This model expands on Proctor’s dimensions and provides a comprehensive framework for understanding the various perspectives involved in the supervisory process [10]. The seven eyes of the model represent seven viewpoints: client, the supervisee’s interventions, the supervisee–client relationship, the supervisee, the supervision relationship, the supervisor, and the wider context. This model emphasizes the importance of considering multiple perspectives and the dynamic nature of a supervisory relationship [10].

Furthermore, the work done by Carroll in 1996 contributed to the understanding of the role of CS in promoting professional development and improving patient outcomes. According to Carroll [9], counseling supervision should encompass a range of essential responsibilities, including consulting, evaluating, and monitoring professional and ethical matters. Carroll emphasizes that emotional awareness and self-assessment are crucial tasks for all counselors as they engage with their clients.

These classical works provide important insights into the dimensions of CS in nursing. They emphasize the need for a comprehensive approach that encompasses administrative support, emotional well-being, educational development, and the consideration of multiple perspectives. By understanding and applying these dimensions, nurses and their supervisors can create a supportive and empowering environment that foster professional growth and enhance the quality of patient care.

The dimensions of CS have a significant role in nurses’ job satisfaction by providing support, guidance, and professional development opportunities [13]. Researchers have shown that effective CS positively impacts nurses’ job satisfaction because it enhances their confidence, competence, and overall well-being [14]. A study [15] was conducted on community mental health nurses, investigating burnout and the impact of effective CS. The study revealed that effective CS was associated with a reduction in burnout, an increase in job satisfaction, and improvements in patient care by providing enhanced staff development opportunities. Numerous studies conducted in different countries have examined the importance of CS in enhancing nurses’ job satisfaction. These nations include Denmark [16], the United States of America (USA) [17], the United Kingdom (UK) [18], Australia [19], and Finland [20]. These studies have identified various positive effects and benefits associated with CS for nurse managers and staff nurses. Additionally, they have identified factors that can influence the effectiveness of CS.

In Saudi Arabia, limited research has been conducted regarding the importance of CS in relation to nurses’ job satisfaction. Almadani [21] conducted a quasi-experimental study to explore the impact of CS on the job satisfaction of 91 primary health-care nurses in Jeddah, Saudi Arabia. The results of the study indicated that within the specific setting examined, CS had the capacity to improve the job satisfaction of experienced primary health-care nurses during a follow-up period of 6 months [21]. As the field continues to develop, more studies are needed to address the significance of CS for nurses’ job satisfaction within the Saudi Arabian context.

Therefore, the primary objective of this systematic review is to collect, assess, and synthesize the current body of evidence, evaluating its reliability and relevance. The aim was to address the existing research gap, to derive meaningful conclusions, inform health-care policymakers in improving nurses’ outcomes, and facilitate the development of well-informed strategies for future research.

Accordingly, we have framed the research question as “To what extent does clinical supervision impact nurses’ job satisfaction in clinical settings?”

## 2. Materials and Methods

### 2.1. Search Strategy and Eligibility Criteria

We conducted a thorough electronic search using four databases, namely, Medline, CINAHL, Cochrane Library, and PsycInfo, in addition to Google Scholar. Including a variety of databases in our research, such as Medline, CINAHL, Cochrane Library, and PsycInfo, ensures that we have access to a wide range of reliable resources that are specific to our fields. Medline is particularly useful for medical research topics due to its comprehensive biomedical literature, while CINAHL offers tailored literature for nursing and allied health research. In addition, the Cochrane Library is renowned for its evidence-based medicine and high-quality systematic reviews. Furthermore, PsycInfo enriches our research with a diverse range of psychological literature. By incorporating these databases into our research, we can improve the quality and scope of our research and ensure that we have access to the most relevant and reliable information available.

We used the PICO framework question in Table 1 to guide the search, which provided the necessary keywords. These keywords encompassed terms such as “registered nurses”, “mental health nurses”, “nurse directors”, “clinical supervision”, “CS*”, “mentorship”, “job satisfaction”, “reducing burnout”, “stress reduction”, and “improve quality care.” By employing Boolean operators like “AND” and “OR”, we combined these search terms effectively. The combination of operators and different search terms yielded a focused search that produced relevant journals aligned with our research question. To ensure accuracy, we applied search limits and filters, allowing us to locate up-to-date studies meeting our criteria. These filters encompassed scholarly and peer-reviewed studies published between January 2010 and May 2023. The decision to focus on articles from 2010 onwards could be grounded in the desire to capture the most recent trends, innovations, and practices in the field, which are more likely to apply to current health-care settings. Thus, we selected journal articles based on predetermined eligibility criteria.

### 2.2. Inclusion and Exclusion Criteria

This systematic review focused on selecting CS studies that examined the effectiveness of supervising qualified nurses or nurse managers, as well as the outcomes associated with such supervision. Various types of studies and evaluations, including quantitative, qualitative, and mixed methods, were considered. The review included publications between January 2010 and May 2023 written in English. Exclusions comprised studies involving health-care professionals other than nurses, those not addressing the specific PICO question, and books and opinion articles.

### 2.3. Search Outcomes

**Figure 1:**
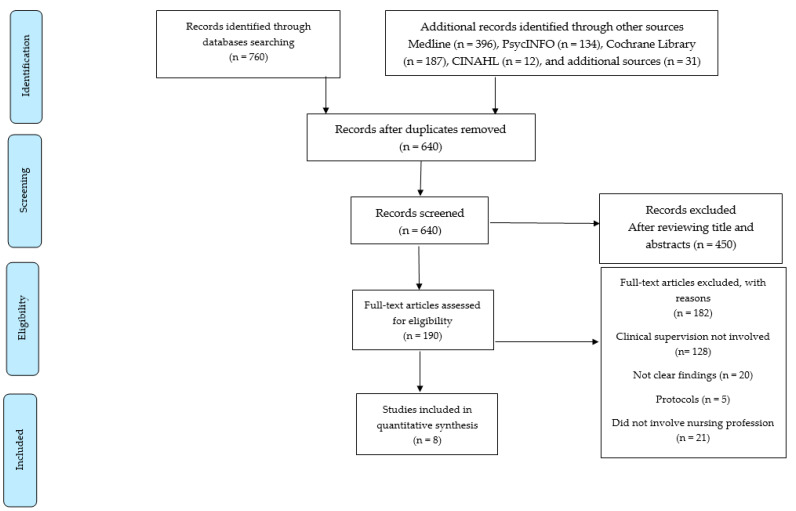
PRISMA flow diagram.

As mentioned above, four databases (PubMed, PsycInfo, Cochrane Library, and CINAHL) were searched, resulting in the identification of 760 articles. The final sample of articles was selected based on the PRISMA flow diagram (Figure 1). All articles were uploaded to the EndNote X9 software (version 9.3.3) and duplicates were removed. The initial screening of titles and abstracts led to the exclusion of irrelevant articles. Out of the 760 full articles, 190 full articles remained; however, 182 articles did not meet the inclusion criteria, resulting in a final sample of 8 articles that were included in the review (as shown in the PRISMA diagram in Figure 1 and Table 2).

### 2.4. Data Extraction

After selecting the eight studies that met the inclusion criteria of this review, the necessary data were extracted using a structured table (see Table 2). The table includes various details such as the author’s name, country, and time frame of the study, as well as the objective, design, sample size, population, method employed, and outcomes of the study. Two evaluators (A.H. (Ali Hudays) and F.G.) independently examined the text of the title and abstract to determine if any relevant citations met the selection criteria. Additionally, two other evaluators (A.H. (Ali Hudays) and A.A.) conducted an electronic search and provided a comprehensive report of all citations that met the predetermined selection criteria. Two evaluators were responsible for extracting and verifying the data. In cases of any discrepancies between the screening of titles, abstracts, or full texts discussed, a third reviewer was consulted, if needed.

### 2.5. Data Synthesis

A narrative synthesis was performed to summarize and evaluate the evidence presented in this review, employing both textual and tabular approaches. The findings are outlined in the subsequent section. In the Discussion section, the results are combined and analyzed.

### 2.6. Appraisal of the Included Studies

This section evaluates a number of studies conducted by different researchers [21,22,23,24,25,26,27,28] that examined CS and its effects on job satisfaction, stress, burnout, and other factors among registered nurses. This review aims to assess the quality of these studies using the nine-point evaluation criteria (Table 3) that can be applied in real-world clinical settings. To evaluate the studies, researchers utilized the Hawker assessment checklist, a tool developed by Hawker et al. in 2002 [29]. This tool comprises nine questions, each of which can be responded to with “good”, “fair”, “poor”, or “very poor.” Upon applying the tool to the research studies, we transformed the responses into a numerical score by allotting 1 point (very poor) to 4 points (good) for each answer. This resulted in a score for each study ranging from a minimum of 9 points to a maximum of 36 points. To determine the overall quality grades, we utilized the following criteria: good quality (A) for scores ranging from 30 to 36 points, medium quality (B) for scores ranging from 24 to 29 points, and low quality (C) for scores ranging from 9 to 24 points. Overall, all the studies included in the review were deemed good quality according to the checklist, as shown in Table 3.

## 3. Results

### 3.1. Study Selection

A total of 640 studies were screened after duplicates were removed. At the title/abstract review stage, 450 out of the 640 were excluded, leaving 190 studies progressing to full-text review, with 8 studies being retained for data analysis and synthesis (Figure 1). The main reasons for exclusion at full-text review were that the publication involved health-care professionals other than nurses and/or did not address the specific PICO question.

### 3.2. Study Characteristics and Quality Assessment

The studies included in this analysis were published between January 2010 and May 2023. They focused on exploring the impact of CS on various factors of job satisfaction, stress, and burnout among registered nurses. The geographical distribution of the studies was as follows: Denmark (two studies), Australia (two studies), and one study each from the United Kingdom, Finland, Saudi Arabia, and Portugal. Among these studies, two were randomized controlled trials [22,23], three were quasi-experimental studies [21,25,26], two others were cross-sectional surveys [27,28], and one was a qualitative study [24]. The sample sizes varied across the studies, ranging from 5 to 139 participants, with an overall total of 1259 participants. The majority of the included studies focused on participants experiencing professional efficacy problems, as highlighted by [22,23,25]. Burnout was also addressed in other studies [26,27]. All the studies comprehensively addressed the interventions and outcomes related to CS. This review investigated various outcome measures associated with CS, including but not limited to its impact on job satisfaction, burnout, stress, patient satisfaction, frequency, and long-term effects of CS, as well as the benefits of reflection and critical thinking in practice and professional development. To evaluate the studies, we utilized the Hawker assessment checklist, a tool developed by Hawker et al. in 2002 [29], which covers various aspects of a study including abstract and title, introduction and aims, method and data, sampling, data analysis, ethics and bias, results, generalizability, and implications and usefulness.

### 3.3. Synthesize the Outcomes

#### 3.3.1. How Does Clinical Supervision Affect Job Satisfaction, Burnout, and Distress Levels?

Three studies [24,25,27] examined the impact of CS on various aspects such as decreasing job dissatisfaction, burnout, and stress, as well as improving the quality of care. One study found that when nurses participated in CS sessions and shared their experiences, it led to a decrease in their psychological distress and an increase in their confidence to discuss personal issues [24]. Another study [27] found a positive correlation between engagement in CS and various positive outcomes, including higher job satisfaction, increased vitality, and improved rational coping, and reduced levels of stress, emotional exhaustion, and depersonalization. Koivu et al. [25] found that nurses who received effective CS reported greater job satisfaction and personal resources, increased motivation, and a higher level of commitment to the organization compared to their colleagues.

#### 3.3.2. The Frequency and Lasting Impacts of Clinical Supervision

Three studies examined the impact of the duration and frequency of CS [21,23,27]. In a study conducted by Almadani [21], it was found that there was a significant improvement in job satisfaction among participants as a result of CS during the 6-month follow-up period. Another study [27] found an association between participation in CS and the experience of positive effectiveness as a result of the frequency of sessions attended by the participants during the last 6 months, while in [23], it was found that the participants in the intervention group showed a considerably higher level of motivation to engage in continuous supervision sessions compared to those in the control group.

#### 3.3.3. The Significance of Reflection in Practical Application

According to Nguyen et al. [30], reflection in the context of learning involves capturing and examining experiences to gain new perspectives. Three studies provided evidence of the existence of such reflective practice [23,24,25]. According to the findings of Cross et al. [24], CS created a secure environment where individuals could openly express and discuss their work-related stress, concerns, and emotions with their fellow supervisees. This supportive setting had a significant role in fostering the growth of reflective practice. According to Gonge and Buus [23], advocating for critical reflection among staff during supervision facilitates their personalized growth and the cultivation of adaptive strategies. In their study, Koivu et al. [25] reached the conclusion that reflective practice, observed among nurses participating in CS, resulted in a notable improvement in professional efficacy and a reduction in psychological distress.

## 4. Discussion

All the studies included in this review assessed the desired outcomes. Among the eight studies, CS was shown to improve the quality of care in one study, enhance job satisfaction in three studies, alleviate burnout in two studies, and reduce stress in two studies. One of the reviewed studies indicated that insufficient CS did not lead to a decrease in burnout. The impact of CS on job satisfaction can be either positive or negative, depending on the specific findings of the systematic review and a plethora of other variables. It is likely that nurses who receive CS would experience an increase in job satisfaction, a decrease in stress levels, and a reduction in burnout if CS is implemented.

### 4.1. How Does Clinical Supervision Affect Job Satisfaction, Burnout, and Distress Levels?

In our review, we identified three studies [24,25,27] that investigated the effects of CS on different factors—reducing job dissatisfaction, burnout, and stress levels—as well as enhancing the quality of care. Clinical supervision not only enhances career obligations and knowledge but also alleviates stress [25] and enhances patient satisfaction [22]. These studies suggest that CS reduces burnout, stress, and improves job satisfaction for nurses [25]. When implemented as regular meetings and team-building activities to promote communication and decision-making, as found by [25], CS fosters a culture of learning in the workplace. It is thus crucial for organizations to provide the necessary facilities and support to ensure employees’ optimal health, consequently reducing or preventing fatigue [30].

To increase job satisfaction, the management of organizations should also give high priority to improving working conditions. Organizations that create a positive and conducive work environment, such as providing comfortable physical surroundings, promoting work–life balance, ensuring safety, and offering necessary resources and support, can contribute to higher job satisfaction among employees [31,32]. Conversely, evidence suggests that high levels of stress and depersonalization contribute to job dissatisfaction. However, nurses can enhance their job satisfaction through effective collaboration and teamwork during CS sessions [23]. Furthermore, according to Cross et al. [24], nurses’ psychological distress can be reduced by sharing experiences in CS sessions, creating a more comfortable environment for problem sharing. Additionally, Heaven et al. [33] stated that nurses who participated in CS training demonstrated enhanced communication skills and responsiveness to patients’ concerns. Considering this evidence, it can be inferred that nursing staff in hospitals—in particular, those who are working in psychiatric facilities—will be able to effectively communicate with patients following CS training.

The importance of CS in managing outcomes such as enhanced patient care, professional development, staff well-being and job satisfaction has not received sufficient focus in the health-care context of Saudi Arabia. Insufficient research conducted in this area may be attributed to factors such as the limited understanding of CS, traditional supervision, and resistance to change by management and nurses themselves. The lack of knowledge in these topics could potentially hinder the exploration and investigation of the significance and impact of CS. Based on our analysis, we discovered that CS has the potential to enhance nursing knowledge and expertise. The findings of our review indicate that professionals who receive CS exhibit greater professional development and improved skills compared to those without supervision [25]. It is noteworthy that four out of eight of the included studies were conducted with nurses who are working in psychiatric and community mental health centers, which reflected the importance of understanding their needs as well as their ability to cope with stress levels in their work environments.

### 4.2. The Frequency and Lasting Impacts of Clinical Supervision

In terms of the frequency and lasting impacts of CS, our review found three studies [21,23,27]. Two of them indicated that CS with frequent sessions once per month lasting from 90–120 min over a 6-month period had a positive effect on nurses’ job satisfaction, stress, emotional exhaustion, and depersonalization. One of the studies [23] indicated that CS with frequent sessions lasting from 60–180 min over a 3-month period showed a considerably higher level of motivation for those in the intervention group to engage in more frequent sessions compared to those in the control group. Nonetheless, it is unclear whether there are established standards for the optimal frequency and duration of CS due to a lack of sufficient evidence in the literature. Edward et al. [15] reported that CS is most effective when conducted for a minimum of 45 min per month. Based on their findings, they recommended that managers and employees allocate at least 1 h of monthly managerial and individual time to supervise and discuss learning and development, as these factors were found to enhance the effectiveness of CS [15].

Previous studies conducted in different countries, including Australia, Denmark, the USA, UK, and Sweden, have examined the CS of nurses in psychiatric hospitals, but they have shown inconsistency in terms of time and frequency of the intervention. The duration and frequency of supervision sessions varied across these studies, ranging from one a week to every 2 months and lasting from 30–180 min [34,35,36,37,38,39]. This inconsistency may be attributed to the lack of standardized guidelines within the field or researchers deciding how often and for how long CS sessions should take place in nursing practice based on their individual perspectives. However, we believe that continuing exploration and examination of this important topic can lead to the development of evidence-based guidelines and best practices, ultimately enhancing the quality of CS and promoting professional growth and development among nurses.

### 4.3. The Significance of Reflection in Practical Application

Regarding the significance of reflection in practical application, our review found three studies that addressed this concern [23,24,25]. In the realm of professional practice, reflection is considered essential for establishing a professional identity. Moreover, it is recognized as a crucial attribute for the growth of independent, critical, and advanced professionals. Various studies, such as those conducted by [23,25], support the notion that reflection has a significant role in professional development. Reflection on practice enables clinicians to delve into the underlying meanings behind their actions and emotional reactions, leading to enhanced cognitive understanding and clinical reasoning. Gonge and Buus [23] argued that encouraging critical reflection among staff during supervision promotes their individualized growth and the development of adaptive strategies. Koivu et al. [25] found that nurses who engaged in reflective practice during CS reported increased professional efficacy, reduced psychological distress, and improved mental health. Consequently, it is crucial to provide all clinical staff with the opportunity to participate in reflective practice to enhance and refine their professional skills and address their well-being.

Furthermore, Koivu et al. [25] observed a significant enhancement in the quality of work, professional effectiveness, and decision-making among individuals who received CS intervention. This intervention has proven to be advantageous and addresses the psychological distress experienced by nurses in the absence of supervision in their work settings [24,25]. Notably, perceptions of leadership support increased, despite no change in burnout levels or workload demands. In a study conducted by Alhawsawi et al. [40], it was suggested that the introduction of CS could be a beneficial strategy for nurses in Saudi Arabia. The researchers recommended that the Saudi Ministry of Health consider developing comprehensive policies and guidelines for implementing CS within health-care systems. These policies should be designed to ensure that nurses possess the necessary knowledge and skills related to CS and the essential best practices necessary to provide quality care for patients and their families. Previous research by Cross et al. [24] has shown that the incorporation of structured CS in a supportive environment can lead to a decrease in burnout and an increase in job satisfaction among nurses.

Moreover, studies [24,25] demonstrated that better CS had a positive impact on various outcomes such as psychological distress, motivation, organizational commitment, and job satisfaction. The quality of supervision was enhanced when supervisors had extensive training, leadership support, and followed CS frameworks. Clinical supervision provided nurses and practitioners with opportunities to explore creative possibilities and gain insights into the challenges presented by different patient cases. Furthermore, it served as a mentorship tool for nurses, reassuring them that they were not alone when facing issues in their clinical practice [24].

Nurses were instructed to express their emotions and thoughts and receive validation from their supervisors when they encountered stressful events in their work environment. Nurses working in mental health facilitates were able to incorporate appropriate responses to critical situations through CS, as shown in studies involving distressed patients [25,27]. Clinical supervision helped nurses effectively handle their distress and alleviate stress, anxiety, and depression caused by work overload [39]. Moreover, nurses became more equipped to confront challenging situations in their daily work, leading to increased confidence in resolving complex medical and psychological issues in the workplace.

Mann et al. [41] described CS as a process aimed at supporting health-care professionals in enhancing their skills and enhancing their ability to handle emergency situations. In the studies examined for this review, the importance of patient well-being and safety emerged as crucial aspects of CS in the nursing field [22].

### 4.4. Strengths and Weaknesses

The review encompassed studies conducted in various nations. The inclusion of studies from multiple countries allows for an exploration of clinical supervision practices in diverse health-care environments and cultural backgrounds. By incorporating a range of countries, the results can account for differences in health-care systems, cultural values, and policies, thereby enhancing the applicability of the findings to a wider global context. Also, the review encompassed various types of studies that could enhance the findings and contribute to a more comprehensive understanding of the research topic. In addition, the majority of the studies had larger samples, which could potentially increase the applicability of the findings. According to [42], utilizing a larger sample than necessary can improve the accuracy of the results and make them more representative of the population. Furthermore, a significant majority of research studies have explicitly indicated the quantity of CS sessions, showcasing a high level of methodological transparency. This transparency is of utmost importance when assessing the credibility and dependability of a study. By disclosing the extent and duration of the intervention, it enables readers to gain a comprehensive understanding of the study’s design and implementation.

In terms of weaknesses, the review included studies that might have threats to external validity as a result of low response rate. According to [43], if only 20% of people respond to a survey, the remaining 80% can introduce nonresponse bias. As a result, the data quality is impacted, leading to a decrease in the generalizability of study results. Furthermore, a significant number of research studies failed to provide comprehensive information regarding the determination of sample size. This includes the absence of details on the utilization of G-Power software and the specific parameters employed to identify the appropriate sample size. Consequently, the distribution of survey questionnaires to participants without any prior calculations or justification of sample size raises serious doubts about the study’s methodological rigor [44]. Furthermore, the majority of studies included in the review were based on convenience sampling rather than equal probability sampling. This approach may introduce bias into the sample, potentially compromising the validity and generalizability of the findings.

### 4.5. Limitations

Despite the valuable insights provided by the current review, it is important to acknowledge several limitations that may impact the generalizability and applicability of the conclusions. Firstly, the inclusion of merely eight studies restricts the generalizability of our findings. A broader search encompassing a larger number of studies, particularly from diverse countries and health-care settings, would significantly enrich the research’s comprehensiveness and enhance its applicability. Secondly, the temporal limitation of our study may be considered a weakness, as it only focuses on data up until mid-2023. Future research should aim to include more recent studies to capture any potential developments or changes in the field. Furthermore, the scope of the review was restricted to papers written in English, which means that studies conducted in other languages were excluded. Consequently, there is a possibility that relevant articles in different languages were not considered. Additionally, due to the time constraints and the limited coverage of the review using only four databases, it is possible that other significant studies were missed.

### 4.6. Implications

Adequate clinical supervision plays a crucial role in ensuring the delivery of high-quality health-care services in various health-care environments. Opportunities exist to improve the policy and strategies in response to clinical supervision by providing continuous education and clinical practice. To achieve this, we recommend that the Ministry of Health in Saudi Arabia take specific actions, firstly by establishing comprehensive policies and guidelines that clearly define the expectations and requirements for clinical supervision. These guidelines will serve as a roadmap for supervisors, ensuring consistency and maintaining the quality of their practices. Secondly, invest in training and education programs for supervisors. By equipping them with the necessary skills and knowledge, they will be better prepared to provide effective supervision to health-care professionals. These programs should focus on enhancing health-care professionals’ leadership abilities, communication skills, and clinical expertise. Additionally, it is crucial to support research and innovation in supervision practices. By encouraging research initiatives and promoting innovation, the ministry can stay at the forefront of advancements in clinical supervision. By implementing these strategic recommendations, the Ministry of Health in Saudi Arabia could elevate clinical supervision practices, foster professional development, enhance patient outcomes, and maintain a high level of health-care delivery throughout the country.

## 5. Conclusions

This systematic review examined the impact of CS on various aspects of nurses’ job experience, including job satisfaction, burnout risk, stress levels, and quality of outcomes. The review identified and analyzed three main themes related to the effects of CS. The results suggest that CS is linked to favorable impacts on nurses’ job satisfaction and various health outcomes, including decreased burnout, reduced stress, and enhanced quality of patient care. However, its effectiveness in improving job satisfaction was evident at the 6-month follow-up. This indicates gaps in knowledge regarding the frequency and number of sessions required for the impact of CS on nurses’ job satisfaction and other outcomes and the frequency and number of CS sessions required to achieve positive outcomes. This gap in knowledge presents an opportunity for future research to delve more deeply into this aspect of CS. Further investigation is needed to determine the optimal frequency and number of CS sessions that yield the maximum benefit for nurses. Future research could explore the effects of different session frequencies, such as weekly or monthly sessions, and examine how the duration and intensity of CS interactions influence job satisfaction and other relevant outcomes. Additionally, understanding the variations in CS requirements across different nursing specialties or contexts would contribute to a more nuanced understanding of the topic. By addressing this gap in knowledge, future studies can provide valuable insights into the design and implementation of effective CS programs that enhance nurses’ job satisfaction and overall well-being. Such research is crucial for supporting the development of evidence-based guidelines and practices in clinical supervision, ultimately benefiting both nurses and the health-care organizations they serve.

## Figures and Tables

**Table 1:** Summary of Literature Search Based on Inclusion Criteria.

Inclusion Criteria	Exclusion Criteria	Keywords	Database Searched	Number of Studies
Studies that examined the effectiveness of supervising qualified nurses or nurse managers.	Studies involving health-care professionals other than nurses.	Qualified Nurses OR Registered Nurses OR Mental Health Nurses OR Psychiatric Nurses OR Nurses Managers OR Nurse Directors AND Clinical Supervision OR Clinical Oversight OR Mentorship OR CS* AND Job Satisfaction OR Work Satisfaction OR Reducing Stress OR Reducing Burnout OR Improving quality care.	Medline	396
Various types of studies and evaluations, including quantitative, qualitative, and mixed methods.	Studies not addressing the specific PICO question.		PsycInfo	134
Studies written in English language.	Books.		Cochrane Library	187
Studies published between January 2010 and May 2023.	Opinion articles.		CINAHL	12
	Studies published before 2010.		Google Scholar	31
				Total: 760

**Table 2:** Characteristics of the included studies.

Author Name/Year/Country/Time Frame	Study Aim	Design	Study Sample	Methodology Process	Number of Sessions/Frequency/Duration	Outcomes
		**RCT (Two Studies)**				
[22] Australia Between July 2007 and January 2009	To investigate the effectiveness of clinical supervision (CS) in mental health settings in Queensland, Australia, and to contribute to the evidence base regarding the relationship between CS and quality of care and patient outcomes in mental health nursing.	A randomized controlled trial.	At the beginning of the study, the intervention group consisted of 139 Community Mental Health Nurses, 24 Clinical Supervisor Trainees who participated in the 4-day Clinical Supervision Course, 115 Supervisors, 82 Patients, and 43 Unit Staff. These participants were recruited from nine different tertiary care hospital sites. The control group included 71 Community Mental Health Nurses, 88 Patients, and 11 Unit Staff who were selected randomly over a period of 12 months. Among the 24 supervisors, 17 were female, accounting for 71% of the group. Two supervisors withdrew from the study after completing the Clinical Supervision course. Internal Validity Concern: Purposive sampling There is no report of power analysis.	A semi-structured interview was carried out with a senior nursing manager and other non-participating members of the nursing staff, totaling 17 individuals. Thematic content analysis was employed to analyze the data obtained from monthly diaries collected from 24 trainees, amounting to a total of 139 entries. For the assessment of mental health nurses’ performance, a quantitative tool was utilized both at baseline and after one year of continuous service. This tool comprised various measures, including the general health questionnaire, Manchester clinical supervisory scale (MCSS), Maslach burnout inventory (MBI), short form health survey, nursing work index, and mental health problems perception questionnaire. Internal Validity Concern: Reliability and validity of instruments did not report. External Validity Concern: Response rate: Not reported.	Once a month for 12 months.	1. Participants in the CS courses and supervisory training agreed that taking part in them had a positive impact on their workplace. 2. 139 CS trainee diary accounts were analyzed along with qualitative data collected from 17 non-involved participants.
[23] Denmark January 2012–February 2013	To test the effects of a meta-supervision intervention in terms of participation, effectiveness, and benefits of clinical supervision of psychiatric nursing staff.	A randomized controlled trial.	A total of 83 nursing staff members working in three general psychiatric wards were randomly assigned to either an intervention group (*n* = 40) or a control group (*n* = 43). Internal Validity Concern: Randomization sampling There is no report of power analysis.	Efficacy and benefits of clinical supervision were assessed at baseline using self-reported questionnaires. A prospective study of clinical supervision participation was conducted over a period of three months following the intervention. External Validity Concern: Response rate at baseline 77.1%, and in the final statistical analysis was 55.4%.	-10 sessions. -For three months. -lasted on average 100 min (range 60–180 min)	Compared to individuals in the control group, participants in the intervention group showed a considerably higher level of motivation for workers to engage in continuous supervision sessions. Promoting critical reflection among staff during supervision fosters their personal growth and facilitates the development of adaptive strategies tailored to their individual needs.
		Qualitative Study (One Study)				
[24] February 2006–June 2006	To address the high stress, depersonalization, burnout, and job dissatisfaction often experienced by nursing managers through the use of clinical supervision as a strategy to reduce these issues.	Qualitative exploratory study.	The study included a focus group of six associate nurse unit managers in a busy medical ward of a tertiary teaching hospital, and four staff members (66%) who participated in the study.	Focus group interview.	-15 sessions. -An hour weekly. -For six months.	The nurses’ psychological distress was reduced and their confidence in sharing their issues increased when they shared their experiences in CS sessions.
		Quasi-experimental studies (three studies)				
[21] Saudi Arabia Between March 2016 and October 2016	To investigate whether clinical supervision improves job satisfaction for qualified nurses in primary health care in Jeddah, Saudi Arabia.	Quasi-experimental design (non-equivalent control group pre-test and post-test).	The study comprised (*n* = 91) nurses from six selected PHC centers in Jeddah, SA. care, for the intervention (*n* = 43) and non-intervention (*n* = 48). Internal Validity Concern: - Sample size: There is report of power analysis. 80%. -Convenience sampling.	1- MSQ (Minnesota Satisfaction Questionnaire): Cronbach’s alpha = 0.85–0.91 2- Semi-structured interview. Internal Validity Concern: -Instrumentations: Not a problem. External Validity Concern: -Response rate = 85%.	-Once per month, with each session lasting two hours, for a cumulative maximum of 12 h.	The qualitative results indicated that participants in the intervention group experienced higher levels of job satisfaction than those in the control group. From a qualitative point of view, a significant proportion of participants demonstrated enhanced job satisfaction after 6 months of clinical supervision. The findings of this study indicate that, in this particular context, clinical supervision has the potential to enhance the job satisfaction of qualified primary health care nurses. This conclusion is drawn from the observation that job satisfaction improved after a six-month follow-up period.
[25] Finland Between 2003 and 2007	To investigate the impact of clinical supervision (CS) on the enhancement of well-being among medical-surgical nurses in their workplace.	Quasi experimental study	The study population consisted of medical-surgical nurses working in a Finnish university hospital. The research was conducted over a 4-year period, from 2003 to 2007. A total of 166 nurses participated in the study, and they were divided into three groups: nurses who received effective clinical supervision (n = 41), nurses who found their clinical supervision less effective (n = 43), and nurses who did not attend clinical supervision (n = 82). Questionnaire surveys were used to assess the nurses’ perceptions of work and health. Internal Validity Concern: -There is no report of power analysis. -Convenience sampling.	1. Nordic Questionnaire for Psychological and Social Factors at Work (QPSNordic): Cronbach’s alpha = 0.89 2. General Health Questionnaire (GHQ12): Cronbach’s alpha = 0.90 3. Maslach Burnout Inventory–General Survey (MBI–GS): Cronbach’s alpha = 0.80 4. MCSS: Cronbach’s alpha = 0.97 Internal Validity Concern: -Instrumentations: Not a problem. External Validity Concern: - Response rate: Not reported.	Not stated.	The results showed that nurses who received efficient clinical supervision reported having more job and personal resources, higher motivation, and a stronger commitment to the organization compared to their peers. However, they scored lower on the dimension of professional inefficacy, which is a component of burnout, compared to other nurses. This suggests that clinical supervision can be seen as an additional job resource that contributes to overall well-being at work. Efficient clinical supervision may serve as both an antecedent and a consequence of well-being in the workplace. The study concluded that the advantages of clinical supervision included positive outcomes such as enhanced professional autonomy and self-efficacy, which were achieved through critical reflection and transformative learning.
[26] Portugal The study was developed from 2017 to 2019	To assess the impact of implementing the SafeCare clinical supervision model on the job satisfaction of nurses and their emotional competence profile.	A quasi-experimental study (pre-test post-test type, with no control group).	The study was aimed to recruit 44 nurses or expert nurses, but for some reasons such as absenteeism related to unsafe pregnancy (*n* = 6; 13,6%), service transfer (*n* = 4; 9,1%), health institution relocating (*n* = 3; 6,8%), and long-term sickness absenteeism (*n* = 3; 6,8%), the study only completed by 28 nurses. Internal Validity Concern: -There is no report of power analysis. -Convenience sampling.	1. Job Satisfaction Scale: Cronbach’s alpha = 0.96 2. Veiga Emotional Competence Scale: Cronbach’s alpha = 0.94 3. Descriptive statistical analysis and the Wilcoxon Test were conducted. Internal Validity Concern: -Instrumentations: Not a problem. External Validity Concern: - Response rate: Not reported.	Not stated.	The results indicate that there is no noteworthy difference in the job satisfaction of nurses between the pre-test and the post-test, as the *p*-value is 0.12. The emotional competence profile of nurses did not exhibit any significant statistical differences before and after the intervention, as indicated by a *p*-value of 0.93. The research findings indicate that the SafeCare Model requires enhancements, proposing an increase in the duration of training provided to nurses and reinforcing the health-care facility’s connection to the Model.
		Cross-sectional surveys (Two Studies)				
[27] Denmark 2011	To investigate the associations between participation in clinical supervision, its effectiveness, and various benefits such as job satisfaction, vitality, coping, and reduced stress and burnout.	Quantitative Cross-sectional survey.	This study consisted of 136 nursing staff members in permanent employment. They were selected from nine general psychiatric wards and four community mental health centers at a Danish psychiatric university hospital. 145 of 239 people responded, which is a moderate response rate (60.7%).	1. Demographic background. 2. MCSS: Cronbach’s alpha = 0.86 3. Coping Styles Questionnaire (CSQ): Cronbach’s alpha = 0.81 4. Copenhagen Psychosocial Questionnaire (COPSOQ) scales: On stress the Cronbach’s alpha = 0.81 On job satisfaction Cronbach’s alpha = 0.83 Internal Validity Concern: Instrumentations: Not a problem. External Validity Concern: Response rate = 60.7%.	-Number of sessions: N/S -Group sessions were the format used for all the clinical supervision sessions. Additionally, the most common duration for these sessions was 90 min.	The outcomes of the study suggested that participation in clinical supervision was associated with the effectiveness of clinical supervision, as measured by the Manchester Clinical Supervision Scale (MCSS). Higher MCSS scores were associated with various benefits, including increased job satisfaction, vitality, rational coping, and reduced stress, emotional exhaustion, and depersonalization. The study supported the proposed model for investigating the benefits of clinical supervision, highlighting the importance of participation, effectiveness, and individual and workplace factors in determining the positive outcomes of clinical supervision in psychiatric nursing.
[28] UK Between April 2014 and June 2014	To explore burnout, the perceived effectiveness of clinical supervision, and the ward environment in a medium secure forensic psychiatric unit in the United Kingdom. The study aimed to investigate the predictors of burnout in this setting, specifically examining the relationship between burnout and factors such as support and the ward environment.	A cross-sectional design.	The sample population of this study consisted of nursing staff working in a medium secure forensic psychiatric unit in the United Kingdom. The total number of participants in the study was 137. Internal Validity Concern: -Convenience sampling.	1. Manchester Clinical Supervision Scale, version 26 (MCSS−26): Cronbach’s alpha = 0.92 2. English Essen Climate Evaluation Schema (EssenCES): Cronbach’s alpha = 0.84 3. Maslach Burnout Inventory—Human Services Survey (MBI-HSS): Cronbach’s alpha = 0.90 Internal Validity Concern: -Instrumentations: Not a problem. External Validity Concern: -Response rate = 87.42%.	Not stated.	The study revealed that approximately 10% of the nursing staff encountered burnout. Age and the hospital ward’s working environment emerged as significant factors that strongly influenced burnout. Interestingly, clinical supervision showed only a minimal correlation with reducing burnout. These findings raise concerns about the effectiveness of clinical supervision as an intervention for burnout. Instead, the study proposes that interventions should focus on establishing a positive and supportive environment within the wards. Furthermore, the study emphasized the vulnerability of younger nursing staff to burnout, underscoring the necessity of tailored support and resources for their well-being.

Abbreviations: CS: clinical supervision; RCT: randomized controlled trial; MCSS: Manchester Clinical Supervisory Scale; MBI: Maslach Burnout Inventory; QPSNordic: Nordic questionnaire for psychological and social factors at work; GHQ12: General Health Questionnaire; MBI-GS: Maslach Burnout Inventory—General survey; MSQ: Minnesota Satisfaction Questionnaire; CSQ: Coping Styles Questionnaire; COPSOQ: Copenhagen Psychosocial Questionnaire Scale; MCSS-26: Manchester Clinical Supervision Scale, version 26; EssenCES: Essen climate evaluation schema; MBI-HSS: Maslach Burnout Inventory—Human services survey.

**Table 3:** Hawker’s assessment tool (level of evidence).

Author	Abstract/Title	Introduction/Aims	Method/Data	Sampling	Analysis	Ethics/Bias	Results	Transferability/Generalizability	Implications	Total	Grade
[22]	4	4	2	2	4	4	4	3	4	31	Good (A)
	A well-organized summary containing comprehensive details and a concise heading.	Provide concise background and state the aims of the study clearly.	The provided explanation on the methodology for data gathering lacks comprehensiveness. Although a detailed description of the instruments used has been provided, there is no mention or reporting of the reliability and validity of these instruments.	While sampling is mentioned, there are limited specific descriptive details provided.	A concise explanation of the analysis methodology employed is provided, along with a description of how the themes were derived.	The matters concerning confidentiality and consent were duly acknowledged and dealt with.	There is an ample amount of data available to substantiate the findings.	While certain aspects of the context and setting are outlined, additional information is required to facilitate replication or comparison of the study with others.	They proposed a clinical implication.		
[23]	4	4	4	2	4	4	4	3	4	33	Good (A)
	The title is clear, and the abstract is well-structured.	The introduction was presented clearly and succinctly, and the statement of aims was clear.	The description was presented clearly and explicitly.	The description of the recruited participants in the study is satisfactory. However, there is a lack of reporting regarding power analysis.	The process of data analysis was clearly outlined.	Ethical considerations and potential biases were appropriately acknowledged and dealt with.	There is an ample amount of data available to substantiate the findings.	It is necessary to compare the described setting with other studies.	They put forward a clinical implication.		
[24]	4	4	4	2	4	2	4	2	4	30	Good (A)
	The abstract is well-organized, containing comprehensive information and a clear title.	Present a succinct background and explicitly state the objectives of the study.	Provide precise information regarding data collection and recording procedures.	Although sampling is mentioned, there is a lack of descriptive details.	Explain how the themes were derived in a clear manner.	Briefly address the issues at hand.	The results directly align with the stated aims of the study.	The description of the context or setting is minimal.	Offer suggestions for future research ideas”.		
[21]	4	4	4	4	4	4	4	4	4	36	Good (A)
	The abstract is well-organized, containing comprehensive information and a clear title.	The discussion is succinct in its back-ground. Furthermore, the study has identified areas of knowledge that are lacking.	The selected approach is appro-priate and has been clearly elucidated.	The sample size used in the study was justified.	There is a clear description of the data analysis methodology.	The issues of confidentiality and consent were appropri-ately addressed.	The findings are explicitly stated, easy to com-prehend, and follow a logical progression. The results are effectively explained in both the text and tables.	The context and set-ting of the study are adequately described, allowing for mean-ingful comparisons with other contexts and settings.	The study suggests practical implications.		
[25]	4	4	4	2	4	4	4	4	4	34	Good (A)
	The abstract is well-structured, containing com-plete infor-mation and a clearly defined title.	The background of the discussion is concise. Furthermore, the study identified gaps in knowledge.	The chosen methodology is suitable and explained clearly.	Sampling mentioned but few descriptive details.	There is a clear description of the data analysis methodology.	The issues of confidentiality and consent were appropri-ately addressed.	The findings are clearly articulated, easily com-prehensible, and presented in a logical se-quence. Additionally, the results are effectively explained both in the text and supported by well-designed tables.	The study provides an adequate description of the context and setting, enabling meaningful comparisons with other con-texts and settings.	Proposes clinical implications.		
[26]	3	4	4	3	3	4	4	4	4	33	Good (A)
	The abstract contains the majority of the necessary information.	Present a clear background with a concise statement of the study objective.	The selected approach is appropriate and has been elucidated with clarity.	The sample size is adequately justified. While most information is provided, some details such as race and context are missing. Moreover, there is no report of power analysis.	The analysis is discussed in a descriptive manner.	The issues of confidentiality and consent were appropriately addressed.	The findings are explicitly stated, easy to understand, and follow a logical progression. The results are effectively explained in both the text and tables.	The context and setting of the study are described sufficiently to allow for comparisons with other contexts and settings.	The study suggests ideas for further research.		
[27]	4	4	4	4	4	4	4	4	4	36	Good (A)
	The abstract is well-structured, containing comprehensive information and a clear title.	Present a clear background along with a concise statement of the study objective.	The chosen methodology is suitable and explained clearly.	Detailed information regarding the participants and their recruitment process was provided.	There is a clear description of how the analysis was conducted.	The issues of participants’ confidentiality were appropriately addressed.	The findings are explicitly stated, easy to understand, and follow a logical progression. The results are effectively explained in both the text and tables.	The context and setting of the study are described sufficiently to allow for comparisons with other contexts and settings.	The study suggests practical implications for practice.		
[28]	3	4	4	4	4	4	4	4	4	35	Good (A)
	The abstract incorporates most of the essential information.	Offer a concise background and clearly state the aims of the study.	The chosen methodology is suitable and explained clearly.	Detailed information regarding the participants and their recruitment process was provided.	There is a clear description of how the analysis was conducted.	The issues of confidentiality and consent were appropriately addressed.	The findings are explicitly stated, easy to understand, and follow a logical progression. The results are effectively explained in both the text and tables.	The context and setting of the study are described sufficiently to allow for comparisons with other contexts and settings.	The study suggests ideas for further research.		

## Data Availability

Data are contained within the article.

## References

[1] Milne D. (2007). An Empirical Definition of Clinical Supervision. Br. J. Clin. Psychol..

[2] Couper K., Kimani P.K., Abella B.S., Chilwan M., Cooke M.W., Davies R.P., Field R.A., Gao F., Quinton S., Stallard N. (2015). The System-Wide Effect of Real-Time Audiovisual Feedback and Postevent Debriefing for In-Hospital Cardiac Arrest: The Cardiopulmonary Resuscitation Quality Improvement Initiative*. Crit. Care Med..

[3] Martino S., Paris M., Añez L., Nich C., Canning-Ball M., Hunkele K., Olmstead T.A., Carroll K.M. (2016). The Effectiveness and Cost of Clinical Supervision for Motivational Interviewing: A Randomized Controlled Trial. J. Subst. Abuse Treat..

[4] Wolfe H., Zebuhr C., Topjian A.A., Nishisaki A., Niles D.E., Meaney P.A., Boyle L., Giordano R.T., Davis D., Priestley M. (2014). Interdisciplinary ICU Cardiac Arrest Debriefing Improves Survival Outcomes*. Crit. Care Med..

[5] Accurso E.C., Taylor R.M., Garland A.F. (2011). Evidence-Based Practices Addressed in Community-Based Children’s Mental Health Clinical Supervision. Train. Educ. Prof. Psychol..

[6] Dorsey S., Pullmann M.D., Kerns S.E.U., Jungbluth N., Meza R., Thompson K., Berliner L. (2017). The Juggling Act of Supervision in Community Mental Health: Implications for Supporting Evidence-Based Treatment. Adm. Policy Ment. Health.

[7] Rothwell C., Kehoe A., Farook S.F., Illing J. (2021). Enablers and Barriers to Effective Clinical Supervision in the Workplace: A Rapid Evidence Review. BMJ Open.

[8] AhnAllen C., Karel M., Topor D. (2013). Developing as a Clinical Supervisor: Competence, Style, and Self-Awareness. MedEdPORTAL J. Teach. Learn. Resour..

[9] Carroll M. (1996). Counselling Supervision: Theory, Skills and Practice.

[10] Hawkins P., Shohet R. (1989). Supervision in the Helping Professions: An Individual, Group, and Organizational Approach.

[11] Proctor Enabling and Ensuring: Supervision in Practice. https://scholar.google.com/scholar_lookup?title=Enabling%20and%20ensuring%3A%20supervision%20in%20practice&pages=21-34&publication_year=1986&author=Proctor%2CB.

[12] Brunero S., Stein-Parbury J. (2008). The Effectiveness of Clinical Supervision in Nursing: An Evidenced Based Literature Review. Aust. J. Adv. Nurs..

[13] Vasan A., Mabey D.C., Chaudhri S., Brown Epstein H.-A., Lawn S.D. (2017). Support and Performance Improvement for Primary Health Care Workers in Low- and Middle-Income Countries: A Scoping Review of Intervention Design and Methods. Health Policy Plan..

[14] Abdullah Mohamed H., Abdelbaset Hamed Awad S., ELsaid ELsabahy H. (2021). The Effect of a Clinical Supervision-Enhancing Strategy for Head Nurses on Their Professional Identity. Egypt. J. Health Care.

[15] Edwards D., Burnard P., Hannigan B., Cooper L., Adams J., Juggessur T., Fothergil A., Coyle D. (2006). Clinical Supervision and Burnout: The Influence of Clinical Supervision for Community Mental Health Nurses. J. Clin. Nurs..

[16] Buus N., Delgado C., Traynor M., Gonge H. (2018). Resistance to Group Clinical Supervision: A Semistructured Interview Study of Non-Participating Mental Health Nursing Staff Members. Int. J. Ment. Health Nurs..

[17] Borders L.D. (2014). Best Practices in Clinical Supervision: Another Step in Delineating Effective Supervision Practice. Am. J. Psychother..

[18] Bishop V. (2008). Clinical Governance and Clinical Supervision: Protecting Standards of Care. J. Res. Nurs..

[19] Mills J.E., Francis K.L., Bonner A. (2005). Mentoring, Clinical Supervision and Preceptoring: Clarifying the Conceptual Definitions for Australian Rural Nurses. A Review of the Literature. Rural Remote Health.

[20] Hyrkäs K., Appelqvist-Schmidlechner K., Paunonen-Ilmonen M. (2002). Expert Supervisors’ Views of Clinical Supervision: A Study of Factors Promoting and Inhibiting the Achievements of Multiprofessional Team Supervision. J. Adv. Nurs..

[21] Almadani Does Clinical Supervision Improve Job Satisfaction for Qualified Nurses in Primary Health Care in Jeddah, Saudi Arabia?—ProQuest. https://www.proquest.com/docview/2411468152?pq-origsite=gscholar&fromopenview=true.

[22] White E., Winstanley J. (2010). A Randomised Controlled Trial of Clinical Supervision: Selected Findings from a Novel Australian Attempt to Establish the Evidence Base for Causal Relationships with Quality of Care and Patient Outcomes, as an Informed Contribution to Mental Health Nursing Practice Development. J. Res. Nurs..

[23] Gonge H., Buus N. (2015). Is It Possible to Strengthen Psychiatric Nursing Staff’s Clinical Supervision? RCT of a Meta-Supervision Intervention. J. Adv. Nurs..

[24] Cross W., Moore A., Ockerby S. (2010). Clinical Supervision of General Nurses in a Busy Medical Ward of a Teaching Hospital. Contemp. Nurse.

[25] Koivu A., Saarinen P.I., Hyrkas K. (2012). Does Clinical Supervision Promote Medical–Surgical Nurses’ Well-Being at Work? A Quasi-Experimental 4-Year Follow-up Study. J. Nurs. Manag..

[26] Rocha I.A.d.R.e.S., Pinto C.M.C.B., Carvalho A.L.R.F. (2021). Impact of Clinical Supervision on Job Satisfaction and Emotional Competence of Nurses. Rev. Bras. Enferm..

[27] Gonge H., Buus N. (2011). Model for Investigating the Benefits of Clinical Supervision in Psychiatric Nursing: A Survey Study. Int. J. Ment. Health Nurs..

[28] Berry S., Robertson N. (2019). Burnout within Forensic Psychiatric Nursing: Its Relationship with Ward Environment and Effective Clinical Supervision?. J. Psychiatr. Ment. Health Nurs..

[29] Hawker S., Payne S., Kerr C., Hardey M., Powell J. (2002). Appraising the Evidence: Reviewing Disparate Data Systematically. Qual. Health Res..

[30] Nguyen Q.D., Fernandez N., Karsenti T., Charlin B. (2014). What Is Reflection? A Conceptual Analysis of Major Definitions and a Proposal of a Five-Component Model. Med. Educ..

[31] Buonomo I., Ferrara B., Pansini M., Benevene P. (2023). Job Satisfaction and Perceived Structural Support in Remote Working Conditions—The Role of a Sense of Community at Work. Int. J. Environ. Res. Public Health.

[32] Zhenjing G., Chupradit S., Ku K.Y., Nassani A.A., Haffar M. (2022). Impact of Employees’ Workplace Environment on Employees’ Performance: A Multi-Mediation Model. Front. Public Health.

[33] Heaven C., Clegg J., Maguire P. (2006). Transfer of Communication Skills Training from Workshop to Workplace: The Impact of Clinical Supervision. Patient Educ. Couns..

[34] Berg A., Hansson U.W., Hallberg I.R. (1994). Nurses’ Creativity, Tedium and Burnout during 1 Year of Clinical Supervision and Implementation of Individually Planned Nursing Care: Comparisons between a Ward for Severely Demented Patients and a Similar Control Ward. J. Adv. Nurs..

[35] Berg A., Hallberg I.R. (1999). Effects of Systematic Clinical Supervision on Psychiatric Nurses’ Sense of Coherence, Creativity, Work-Related Strain, Job Satisfaction and View of the Effects from Clinical Supervision: A Pre-Post Test Design. J. Psychiatr. Ment. Health Nurs..

[36] Hallberg I.R. (1994). Systematic Clinical Supervision in a Child Psychiatric Ward: Satisfaction with Nursing Care, Tedium, Burnout, and the Nurses’ Own Report on the Effects of It. Arch. Psychiatr. Nurs..

[37] Kavanagh D.J., Spence S.H., Strong J., Wilson J., Sturk H., Crow N. (2003). Supervision Practices in Allied Mental Health: Relationships of Supervision Characteristics to Perceived Impact and Job Satisfaction. Ment. Health Serv. Res..

[38] Severinsson E.I., Kamaker D. (1999). Clinical Nursing Supervision in the Workplace--Effects on Moral Stress and Job Satisfaction. J. Nurs. Manag..

[39] Long C.G., Harding S., Payne K., Collins L. (2014). Nursing and Health-Care Assistant Experience of Supervision in a Medium Secure Psychiatric Service for Women: Implications for Service Development. J. Psychiatr. Ment. Health Nurs..

[40] Alhawsawi A.O., Alahmdi A.M., Aly S.M. (2022). Nurse Manager’s Perception about Clinical Supervision at Madinah Hospitals in Saudi Arabia. Nurs. Commun..

[41] Mann K., Gordon J., MacLeod A. (2009). Reflection and Reflective Practice in Health Professions Education: A Systematic Review. Adv. Health Sci. Educ. Theory Pract..

[42] Andrade C. (2020). Sample Size and Its Importance in Research. Indian J. Psychol. Med..

[43] Fincham J.E. (2008). Response Rates and Responsiveness for Surveys, Standards, and the Journal. Am. J. Pharm. Educ..

[44] Gray J., Grove S.K., Sutherland S. (2017). Burns and Grove’s the Practice of Nursing Research: Appraisal, Synthesis, and Generation of Evidence.

